# Suicidal ideation and suicide attempts among high-school students reporting substance use in Northwest Ethiopia: a multischool cross-sectional study

**DOI:** 10.1016/j.abrep.2026.100755

**Published:** 2026-10-02

**Authors:** Getasew Kibralew, Girum Nakie, Tesfaye Segon

**Affiliations:** a Department of Psychiatry, College of Medicine and Health Science, Injibara University, Injibara, Ethiopia; b Department of Psychiatry, College of Medicine and Health Science, University of Gondar, Gondar, Ethiopia

**Keywords:** Suicide, Substance use, Substance-using high-school students, Ethiopia

## Abstract

**Background:**

Substance use and suicidal behavior among high-school students are important public health concerns. However, evidence on suicidal behavior among substance-using high-school students in Ethiopia is limited. This study assessed the prevalence and associated factors of suicidal ideation (SI) and suicide attempts (SA) among substance-using high-school students in Northwest Ethiopia.

**Methods:**

A multicenter cross-sectional study was conducted from 6 January to 14 February 2025 among 405 substance-using high-school students selected from 843 high-school students through multistage sampling. Suicidal behavior and associated factors were assessed using standardized instruments. Bivariable and Firth penalized multivariable logistic regression analyses were performed.

**Results:**

The prevalence of suicidal ideation (SI) and suicide attempts (SA) was 7.4% (95% CI: 4.9–9.8) and 4.4% (95% CI: 2.5–6.6), respectively. SI was associated with depression symptoms (AOR = 2.34, 95% CI: 1.54–9.89), family history of suicide (AOR = 3.78, 95% CI: 2.12–8.52), and khat chewing (AOR = 1.86, 95% CI: 1.01–4.29). SA was associated with female sex (AOR = 2.21, 95% CI: 1.03–10.12), family history of suicide (AOR = 2.46, 95% CI: 1.43–4.67), chronic medical illness (AOR = 3.60, 95% CI: 1.13–7.25), and stress (AOR = 3.07, 95% CI: 1.18–7.54).

**Conclusion:**

Suicidal ideation and suicide attempts were reported among substance-using high-school students. Several socio-demographic, clinical, and substance-related factors were associated with suicidal behavior, highlighting the need for further longitudinal research.

## Highlights

• Substance use and suicidal behavior are important public health concerns among high-school students.
• Among 843 high-school students, 405 met the study definition of substance use and constituted the final analytic cohort.
• Suicidal ideation and suicide attempts were 7.4% and 4.4%, respectively, among substance-using high-school students.
• Suicidal ideation was associated with depression symptoms, family history of suicide, and khat chewing. Suicide attempts were associated with female sex, family history of suicide, chronic medical illness, and stress.
• These findings provide suicidal behavior among substance-using high-school students and support for longitudinal research.

Proposal

## 1 Introduction

The World Health Organization (WHO) reported approximately 800,000 suicide-related deaths globally in 2016. Suicide is a major public-health concern among adolescents and remains one of the leading causes of death among those aged 15–19 years globally (Bachmann, 2018; Bertolote & Fleischmann, 2002). Substance misuse has been consistently associated with elevated suicide mortality. An updated systematic review and meta-analysis of 47 longitudinal studies found a substantially higher risk of suicide among individuals with substance misuse (Athey et al., 2025). Among adolescents, a systematic review and meta-analysis found that recurrent psychotropic recreational drug use and drug misuse were associated with suicidal ideation (Oliveira Gracini et al., 2024). Substance use, particularly alcohol use, has also been associated with suicidal behavior among adolescents. European school-based evidence has shown that alcohol intoxication is associated with an elevated risk of deliberate self-harm among adolescents across several countries (Hibbel, 2024; Tervo-Clemmens et al., 2024). A large Swedish national cohort study also found that alcohol use disorder was associated with increased suicide mortality (Edwards et al., 2024).

Similarly, a cross-sectional study among university students in Uganda found an association between psychoactive substance use and suicidal tendencies (Wesonga et al., 2021). The association between substance use and suicidal behavior is further supported by a meta-analysis of 43 studies involving 870,967 participants (Poorolajal et al., 2016). Adolescents who reported polysubstance use were three to five times more likely to experience suicidal thoughts than those who did not report polysubstance use (Yang, 2024). A pooled population-based analysis of 211,022 adolescents from 58 countries found that, among alcohol users, the prevalence of suicidal ideation and suicide attempts was 40.6% and 41.7%, respectively, among boys, and 42.7% and 42.7%, respectively, among girls (Chen et al., 2025). According to a meta-analysis conducted in Iran, drinking alcohol excessively increases the likelihood of having suicidal thoughts and attempts, as well as being a major contributor to premature death (Darvishi et al., 2015). In western Iran, a study of substance use disorders found that 37.2% of alcohol users reported suicidal ideation and 33.8% had a history of suicide attempts, with alcohol consumption significantly associated with suicide attempts (Farnia et al., 2025).

Suicide remains a major cause of premature mortality among adolescents and young adults. In Canada, suicide is currently the second leading cause of death among people aged 15–34 years (Mete, 2026).

Suicide attempts were more common among in-school teenagers from seven low- and middle-income African nations who reported substance use. The prevalence of suicide attempts was 21.08% among early alcohol initiators and 13.38% among late initiators, compared to 12.77% among non-users; for other drug use, the corresponding prevalences were 42.80%, 28.21%, and 13.97% (Li et al., 2021). Ethiopian studies have demonstrated a substantial burden of suicidal behavior among substance-using populations, with suicidal ideation and suicide attempts reported by 39.5% and 18.6% of patients with substance use disorder in Addis Ababa, respectively (Kassie et al., 2022). In northern Ethiopia, 41.4% of individuals with problematic substance use in South Gondar reported suicidal behavior during the preceding 12 months (Legas et al., 2020), while among substance-using youth in Northwest Ethiopia, the prevalence of suicidal ideation and suicide attempts was 14.5% and 9.9%, respectively (Melkam et al., 2022a).

Suicidal behavior is a complex phenomenon influenced by multiple biological, genetic, psychological, social, environmental, and situational factors (Amare et al., 2018; Masferrer & Caparrós, 2017; Pompili et al., 2010). Among adolescents, suicidal behavior has been associated with multiple and potentially overlapping factors, including substance use, mood disorders, stressful life events, interpersonal difficulties, low social support, loneliness, and hopelessness (Pompili et al., 2012; Poorolajal et al., 2016). In a large school-based study of 19,067 high-school students, higher levels of social support were associated with significantly lower odds of suicide attempts among students reporting opioid misuse (Agyemang et al., 2022). These factors may occur independently or in combination and may contribute to suicidal behavior through different pathways. Suicidal behavior imposes substantial social, emotional, economic, and public-health burdens on individuals, families, communities, and health systems (Jain et al., 2024). However, previous studies in Ethiopia have largely examined suicidal ideation and suicide attempts among general adolescent and youth populations, with limited evidence specifically addressing substance-using high-school students. Thus, evidence on the prevalence and associated factors of suicidal ideation and suicide attempts among substance-using high-school students in Ethiopia remains limited. Therefore, this study aimed to determine the prevalence and associated factors of suicidal ideation and suicide attempts among substance-using high-school students in Northwest Ethiopia.

## 2 Methods and materials

### 2.1 Study area and population

An institution-based cross-sectional study was conducted from 06 January to 14 February 2025 among high-school students in three woredas of Northwest Ethiopia: North Mecha, South Mecha, and Yilmana Densa. The study area had a total population of 506,932, of whom 254,621 were men. The majority of the inhabitants practiced Ethiopian Orthodox Christianity. There were twelve governmental high schools with a total enrollment of 26,524 students, of whom 8023 were male and the remainder were female. There were no private high schools in the study areas. The source population comprised students enrolled in governmental high schools in the three selected woredas, while the study population consisted of eligible students enrolled in the selected governmental high schools who were present during the data-collection period.

### 2.2 Sample size determination and sampling procedure

The sample size was determined by assuming a single population formula. In the initial step, the prevalence of substance use disorder was taken from a previously published study in Debre Markos town, Ethiopia 52.5% (Melkam et al., 2023). By assuming 95%, confidence interval (CI), and margin error of 5%. As a result, the following calculation is made using the prevalence of substance use:

$$n=\frac{\left(\mathrm{Z\alpha}/2\right)\;2\;x\;p\;\left(1-p\right)}{d^{2}}:n=\frac{\left(1.96\right)2\times 0.525\;x.475}{\left(0.05\right){\left(0.05\right)}^{2}}=383$$

A multistage sampling technique was employed. Because students were selected within sampled schools, potential clustering of students within schools was considered in the sample-size calculation. Accordingly, a design effect of 2 was applied to account for the potential loss of statistical precision associated with clustering, consistent with previous school-based studies among adolescents in Ethiopia (Yirga et al., 2016). The design effect was used only for sample-size determination and was not applied as a variance adjustment in the regression analyses. The initial sample size of 383 was multiplied by 2, yielding 766, and a 10% non-response allowance resulted in a final required sample size of 843. Of the 12 governmental high schools in the three study woredas, seven were randomly selected as the sampled schools. The sample was proportionally allocated to the selected schools according to student enrollment and subsequently distributed proportionally across grades 9–12 based on class size. Students were then selected from each grade using a computer-generated simple random sampling method based on their identification numbers. Because allocation was proportional to student enrollment and selection within schools and grades was random, eligible students had an equal or approximately equal probability of selection. A total of 843 high-school students were assessed for substance use, of whom 405 were identified as substance users and 438 as non-substance users. Because the present study focused on suicidal ideation and suicide attempts among substance-using high-school students, the 405 substance users constituted the final analytical sample (Fig. 1).

**Fig. 1:**
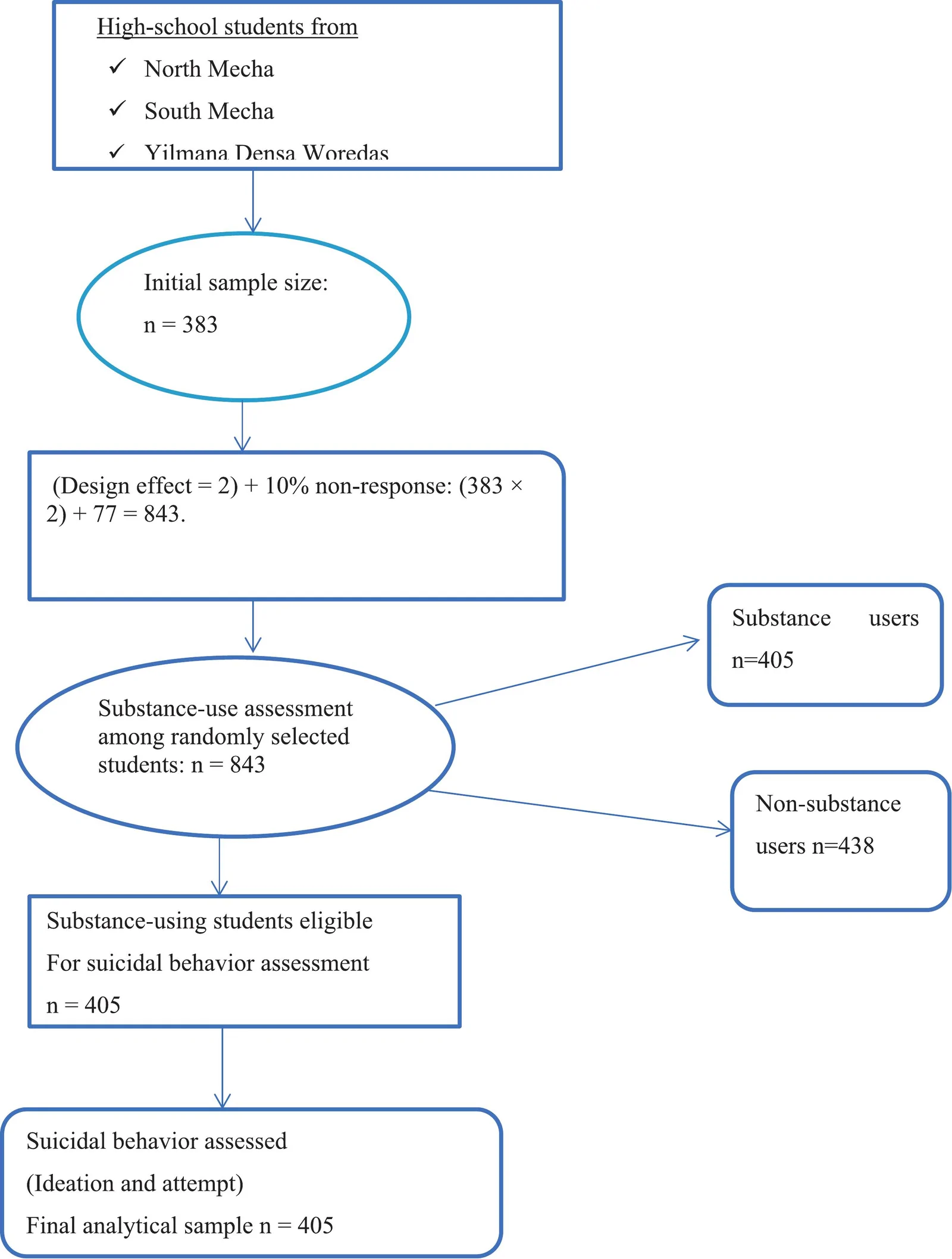
Flow of participant selection and inclusion in the final analytical sample.

### 2.3 Data collection tools

The data were collected using a self-administered questionnaire covering socio-demographic characteristics, substance use, social support, and clinically related factors, including depression, anxiety, and stress. Among the 405 students classified as substance users according to the study definition, suicidal ideation and suicide attempts during the previous 12 months were assessed using the suicidality module of the World Mental Health Survey Initiative version of the World Health Organization Composite International Diagnostic Interview (CIDI). The CIDI suicidality module has been used to assess suicidal ideation and suicide attempts in Ethiopian populations (Gelaye et al., 2013). The module assesses suicidal behaviors over different reference periods. Lifetime suicidal ideation was defined as a “yes” response to the question, “Have you ever seriously thought about committing suicide?”, and lifetime suicide attempt was defined as a “yes” response to the question, “Have you ever attempted suicide?” For the present study, past-year suicidal ideation was defined as reporting serious suicidal thoughts during the previous 12 months, and past-year suicide attempt was defined as reporting an attempted suicide during the previous 12 months. Responses were coded as 1 = yes and 0 = no. The suicidality questions were administered in the self-administered questionnaire to the 405 students who met the study definition of substance use.

Substance use, including alcohol, khat, and cigarette smoking, was assessed using the Alcohol, Smoking and Substance Involvement Screening Test (ASSIST), a World Health Organization–developed and validated instrument (Newcombe et al., 2016). The ASSIST assesses lifetime use and current patterns of substance use and related problems across multiple substance categories. In this study, lifetime substance use was defined as a “yes” response to ASSIST Question 1 for any assessed substance. Participants meeting this criterion were classified as substance users and included in the present analysis. Questions 2–7 were used to assess the frequency and pattern of current use and to generate substance-specific risk scores. For alcohol, ASSIST scores of 0–10, 11–26, and ≥ 27 indicate low, moderate, and high risk, respectively; for other substance categories, scores of 0–3, 4–26, and ≥ 27 indicate low, moderate, and high risk, respectively. Thus, in this study, “substance user” refers to participants reporting lifetime use of at least one assessed substance, whereas current patterns of use and substance-related risk were assessed separately using Questions 2–7 and the corresponding ASSIST risk scores (Newcombe et al., 2016).

The Depression, Anxiety, and Stress Scale (DASS-21) was used to assess depression, anxiety, and stress. The instrument consists of three subscales, each containing seven items. Responses were scored according to the DASS-21 scoring procedure, and each subscale score was multiplied by two before applying the recommended severity cutoffs. Participants with scores ≥10 for depression, ≥8 for anxiety, and ≥ 15 for stress were classified as having depression, anxiety, and stress, respectively (Yeshaw & Mossie, 2017).

Social support was measured by the Oslo-3 social support scale, which ranges from 3 to 14. Those respondents who scored between 3 and 8 were considered to have poor social support, a score of 9–11 was considered to have moderate social support, and a score of 12–14 was considered strong social support (Dalgard et al., 2006).

### 2.4 Data quality control

Data were collected by three BSc nurse professionals using self-administered questionnaires. Three BSc psychiatry professionals also participated by supervising the data collectors. To control the quality of data the questionnaire was initially prepared in English, then translated into the Amharic language, and finally back to English by two language experts and psychiatrists appropriately. The one-day training was given to those data collectors and the supervisor before the actual data collection. Two week before the actual data collection time**,** the questionnaire was pretested on Dangila high school students, which were not included in the main study. A suitable adjustment to the questionnaire was done in response to the feedback from the pre-test.

### 2.5 Data processing and analysis

The collected data were entered using EpiData version 4.6 and exported to SPSS version 25 for analysis. Descriptive statistics, including frequencies and percentages, were used to summarize the study variables. The seven selected schools represented the primary sampling units, with students sampled within schools after proportional allocation across schools and grades. No sampling weights were calculated or applied; therefore, the descriptive statistics and regression analyses were based on unweighted observations. Bivariable binary logistic regression analyses were conducted separately for each independent variable to assess its crude association with suicidal ideation and suicide attempts, and crude odds ratios (CORs) with 95% confidence intervals (CIs) were calculated. The bivariable regression analyses were based on unweighted observations and were not adjusted for clustering at the school level. The design effect of 2 was used only during sample-size determination and was not incorporated into the regression variance estimation.

For the suicidal-ideation and suicide-attempt outcomes, parsimonious multivariable models were specified to minimize the risk of overfitting and unstable estimates given the limited number of outcome events. Candidate predictors were selected based on their clinical and theoretical relevance to suicidal behavior and informed by the bivariable findings, rather than including all variables meeting the *p* < 0.20 criterion. The selected predictors were entered simultaneously into separate multivariable models for each outcome, with the number of fitted parameters considered in relation to the observed number of outcome events. Firth penalized logistic regression was used to reduce small-sample bias and improve the stability of estimates in the presence of limited outcome events. Adjusted odds ratios (aORs) with 95% CIs were reported. There were no missing data for the variables included in either multivariable model; therefore, all 405 participants were included in the complete-case analyses for both suicidal ideation and suicide attempts. The confidence intervals and *p*-values were obtained using the inferential procedure implemented in the Firth penalized logistic regression analysis. All point estimates, confidence intervals, and *p*-values were verified against the corresponding final model output before the results tables were regenerated. Given the limited number of outcome events, the adjusted associations were interpreted cautiously.

## 3 Results

### 3.1 Participant flow

A total of 843 high-school students were approached and participated in the study, yielding a 100% response rate. Of these, 405 met the study definition of substance use and completed the assessment of suicidal ideation and suicide attempts, yielding a 100% response rate for the final analytic sample.

### 3.2 Socio-demographic characteristics of participants

The mean age of the participants was 18.59 ± 1.817 years, ranging from 15 to 25 years. Of the 405 participants, 266 (65.7%) were aged 15–19 years, while 139 (34.3%) were aged ≥20 years. Approximately two-thirds of the participants were male (64.4%), and 297 (73.3%) were originally from rural areas. About one-third of the participants were in grade nine (32.6%), whereas grade twelve represented the smallest proportion (18.0%). The majority of participants were living with both parents (76.5%) (Table 1).

**Table 1:** Sociodemographic characteristics of participants among substance using high school students in Northwest Ethiopia, (*n* = 405).

Variables	Categories	Frequency	Percent
Sex	Male	261	64.4
	Female	144	35.6
Age	≤19	266	65.7
	≥20	139	34.3
Residency	rural	297	73.3
	Town	108	26.7
Living arrangements	Both parents (father and mother	310	76.5
	Single parents (father or mother)	43	10.6
	Alone	22	5.4
	Relatives	30	7.4
Grade	9	132	32.6
	10	95	23.6
	11	105	25.9
	12	73	18.0
Academic performance	≤49.99	34	8.4
	50–74.99	247	61.0
	75–84.99	86	21.2
	≥85	38	9.4
Absenteeism	Yes	44	10.9
	No	361	89.1

### 3.3 Substance and clinical characteristics of participants

Out of the total participants, 170 (42.0%) had depression symptoms, 241 (59.5%) had anxiety symptoms, and 177 (43.7%) students were stressed. Regarding substance use, the majority of the participants 302 (74.57%) were alcohol users whereas khat and cigarette users were 67 (16.54%) and 6,3(8.89%) respectively. Of the participants, 126 (31.1%) had poor social support whereas students who had intermediate and strong social support 157 (38.8%), and 122 (30.1%) respectively (Table 2).

**Table 2:** Clinical and substance-related characteristics of participants among substance-using high school students in Northwest Ethiopia (*n* = 405).

Variables	Categories	Frequency	Percent
Depression	Yes	170	42.0
	No	235	58.0
Anxiety	Yes	241	59.5
	No	164	40.5
Stress	Yes	177	43.7
	No	228	56.3
Having known chronic medical illness	Yes	41	10.1
	No	364	89.9
Family history of suicide	Yes	57	14.1
	No	348	85.9
Substances	Alcohol	302	74.57
	Khat	67	16.54
	Cigarette	36	8.89
Social support	Poor	126	31.1
	Intermediate	157	38.8
	Strong	122	30.1

### 3.4 Prevalence and associated factors of suicidal ideation and attempt

Among 405 students, 30 (7.4%); (95% CI = 4.9–9.8) of the students reported suicidal ideation (SI) during the last twelve-month period and 53 (13.1%) reported a lifetime history of suicidal ideation. The twelve-month prevalence of suicide attempts (SA) was 18 (4.4%); (95% CI = 2.5–6.6) whereas 26 (6.4%) of the respondents had attempted suicide in their lifetime. Twelve (46.1%) respondents had attempted suicide once, 10 (38.5%) had done it twice, and 4 (15.4%) had done so more than two times. Among the 26 participants who reported a lifetime suicide attempt, hanging was the most commonly reported method 14(53.8%), followed by poisoning 10(38.5%).

### 3.5 Factors associated with suicidal ideation

Variables considered for multivariable analysis were selected based on their clinical and theoretical relevance to suicidal ideation and evidence from previous studies among adolescents and young people. Given the limited number of suicidal-ideation events, a parsimonious model was specified to minimize overfitting and model instability. Depression (Takelle et al., 2026; Wang et al., 2024), family history of suicide (Bratu & Moroianu, 2025; Takelle et al., 2026; Tekeba et al., 2025),and khat chewing (Desalegn et al., 2020a) were prioritized for inclusion because they represent psychological, familial, and substance-related factors relevant to suicidal ideation. The final model was estimated using Firth penalized logistic regression to reduce small-sample bias and improve the stability of estimates in the presence of limited outcome events.

In the Firth penalized logistic regression analysis, depression, family history of suicide, and khat chewing were significantly associated with suicidal ideation. Students with depression had higher odds of reporting suicidal ideation than those without depression (aOR = 2.34, 95% CI: 1.54–9.89, *p* = 0.004). Students with a family history of suicide had higher odds of reporting suicidal ideation than those without such a history (aOR = 3.78, 95% CI: 2.12–8.52, *p* = 0.034). Similarly, students who reported khat chewing had higher odds of suicidal ideation than those who did not report khat chewing (aOR = 1.86, 95% CI: 1.01–4.29, *p* = 0.014) (Table 3).

**Table 3:** Bivariable logistic regression and Firth penalized logistic regression analyses of factors associated with suicidal ideation among substance-using high-school students in Northwest Ethiopia (*n* = 405).

Variable	Category	Suicidal ideations		COR (95% CI)	AOR (95% CI)	*P*-value
		Yes	No			
Depression	Yes	19	151	2.56(1.19–5.54)	2.34 (1.54–9.89)	0.004
	No	11	224	1.00	1.00	
Family history of suicide	Yes	13	44	5.75(2.62–12.65)	3.78(2.12–8,52)	0.034
	No	17	331	1.00	1.00	
Khat chewing	Yes	11	72	2.44(1.11–5.35)	1.86 (1.01–4.29)	0.014
	No	19	303	1.00	1.00	

### 3.6 Factors associated with suicide attempts

Variables considered for multivariable analysis were selected a priori based on their clinical and theoretical relevance to suicide attempt and evidence from previous studies among adolescents and young people. Given the small number of suicide-attempt events, a parsimonious model was specified to minimize overfitting and model instability. Female sex (Ferede et al., 2025; Miranda-Mendizabal et al., 2019a), family history of suicide (Ferede et al., 2025; Tekeba et al., 2025), stress (Li et al., 2025), and chronic medical illeness (Aliy et al., 2023) were prioritized for inclusion because they represent demographic, familial, psychological, and physical-health domains relevant to suicidal behavior. The final suicide-attempt model was estimated using Firth penalized logistic regression to reduce small-sample bias and potential separation associated with the limited number of outcome events.

In the Firth penalized logistic regression analysis, female sex, family history of suicide, chronic medical illness, and stress were associated with suicide attempts. Female students had higher odds of reporting a suicide attempt than male students (aOR = 2.21, 95% CI: 1.03–10.12, *p* = 0.023). Students with a family history of suicide had higher odds of reporting a suicide attempt than those without such a history (aOR = 2.46, 95% CI: 1.43–4.67, *p* = 0.007). Similarly, students with chronic medical illness had higher odds of reporting a suicide attempt than those without chronic medical illness (aOR = 3.60, 95% CI: 1.13–7.25, *p* = 0.019). Students experiencing stress also had higher odds of reporting a suicide attempt than those who did not report stress (aOR = 3.07, 95% CI: 1.18–7.54, *p* = 0.008) (Table 4).

**Table 4:** Bivariable logistic regression and Firth penalized logistic regression analyses of factors associated with suicide attempts among substance-using high-school students in Northwest Ethiopia (*n* = 405).

Variable	Category	Suicide attempt		COR (95% CI)	AOR (95% CI)	P-value
		Yes	No			
Sex	Female	11	133	3.00 (1.14–7.92)	2.21 (1.03–10.12)	0.023
	Male	7	254	1.00	1.00	
Family history of suicide	Yes	5	52	2.48 (0.85–7.24)	2.46(1.43–4,67)	0.007
	No	13	335	1.00	1.00	
Chronic medical illness	Yes	6	35	5.03 (1.78–11.22)	3.60 (1.13–7.25)	0.019
	No	12	352	1.00	1.00	
Stress	Yes	13	164	3.54 (1.56–14.88)	3.07 (1.18–7.54)	0.008
	No	5	223	1.00	1.00	

## 4 Discussion

Suicidal ideation and suicide attempts cause serious emotional, physical, and economic impacts. People who attempt suicide and survive may experience serious injuries that can have long-term effects on their health. The finding of the current study showed that the prevalence of suicidal ideation among substance users was found to be 7.4% (95% CI = 4.9–9.8) which was consistent with the findings of other studies done in Nigeria with a prevalence of 6.1% (95% CI: 5.5–6.7) (Adewuya & Oladipo, 2020).

However, the prevalence of suicidal ideation in this study was lower than previous research findings done in Ethiopia was 14.5% (Melkam et al., 2022b). The observed difference may partly reflect differences in study settings, participant characteristics, and the assessment of suicidal ideation. The previous study was conducted at the community level, whereas the present study was conducted in an institutional setting. The other reason could be the difference in the participants' sociodemographic factors between the previous and current studies. The current prevalence of suicidal ideation among substance-using school adolescents was also lower than in research conducted outside of Ethiopia. In South Carolina, suicidal ideation was reported to be 11% (Garrison et al., 1993), and in a comparison study between the USA and China, adolescents' suicidal ideation was 17.4%, and 13.7% respectively (Guo et al., 2021). The possible reason may partly reflect differences in study populations and settings, as the present study specifically focused on substance-using high-school students, whereas the comparison studies included broader adolescent populations. Previous evidence from the United States and China has shown that bullying, feelings of sadness or hopelessness, aggressive behaviors, and specific substance-use behaviors are associated with increased suicidal ideation and suicide attempts. These factors may increase vulnerability to suicidal ideation through greater psychological distress and difficulties in coping with adverse experiences and interpersonal stressors (Kirshenbaum et al., 2024).

The prevalence of suicide attempts among substance use high school students was found to be 4.4% (95% CI = 2.5–6.6) which was Consistent with the findings of other studies done in Nigeria and Canada found to be 2.8% and 4.9% respectively (Adewuya & Oladipo, 2020; Marshall et al., 2013). Similarly, the current study is in line with a study done in a comparative study between the USA and China adolescents which results in 5.7%, and 2.7% respectively (Guo et al., 2021). However, the prevalence of suicide attempts in this study is lower than in a previous study done a community study was done in Ethiopia, South Africa, American youth in New Mexico(American Indians and Alaska Natives) and South Carolina were 9.9%, 14.8%, 12.0% and 14.0% and 7.5% respectively (Agyemang et al., 2022; Garrison et al., 1993; Khuzwayo et al., 2018; Melkam et al., 2022b). The observed differences may partly reflect variation in the sex distribution of participants, as well as differences in study settings and population characteristics. In South Africa, 886 (50.4%) of the participants were female, while 873 (49.6) were male, and in South Carolina, 2063 (55%) of the participants were female, while 1701 (45%) were male (Garrison et al., 1993; Khuzwayo et al., 2018). In contrast, only 144 (35.6%) of the participants in the current study were female, whereas 261 (64.4%) were men. The higher prevalence of suicide attempts among females may partly reflect the greater burden of internalizing symptoms, including depression and anxiety, which have been associated with suicidal behavior among adolescents (Hawton, 2000). In addition, Agyemang et al. examined 19,067 high-school students in New Mexico using repeated cross-sectional, clustered school-survey data and found that higher social support was associated with lower odds of suicide attempts among students reporting opioid misuse (Agyemang et al., 2022). In contrast, the present study included 405 high-school students reporting lifetime use of alcohol, khat, or tobacco/cigarettes, among whom 18 reported a suicide attempt in the previous 12 months. The studies differed in population, sample size, substances assessed, social-support measurement, number of outcome events, and analytical methods, which should be considered when interpreting the findings.

Regarding factors affecting suicidal ideation, students having a family history of suicide were 3.78 times more odds to have suicidal ideation as compared with students who had no family history of suicide. This finding is consistent with other findings done in Egypt, England, and Columbia (Bassiony et al., 2022; Brent & Mann, 2006; Posner et al., 2011). The association between family history of suicide and suicidal ideation may partly reflect a combination of genetic vulnerability and shared familial, psychological, and environmental factors. Familial transmission of suicidal behavior may involve heritable susceptibility, while shared family experiences and environmental stressors may also contribute to vulnerability to suicidal behavior (Rihmer et al., 2013; Willour et al., 2012).

Students who had depression were about 2.34 times to develop suicidal ideation when compared to those who did not. This was supported by a previous study conducted in Nigeria, Egypt, and Spain (Adewuya & Oladipo, 2020; Bassiony et al., 2022; Masferrer et al., 2018). The possible reason could be, that adolescents with depression may have frequent sadness, tearfulness, hopelessness, decreased interest in activities, persistent boredom, withdrawal from friends, low self-esteem, and guilt, and all of these lead to an individual having suicidal thoughts (Galaif et al., 2007).

The odds of having suicidal ideation were nearly four times (AOR = 1.86, 95% CI: 1.01–4.29) more prevalent among Khat chewers as compared with nonchewers. This finding is supported by two different studies in Ethiopia. In general university students (AOR = 1.78, 95% CI 1.05–3.00) and medical students in particular (AOR = 4.46, 95% CI 3.32–10.02), these two investigations showed that khat chewers were 1.8 and 4.5 times more likely to have suicide thoughts respectably (Amare et al., 2018; Dachew et al., 2016; Desalegn et al., 2020b). The observed association between khat chewing and suicidal ideation may partly reflect The observed association between khat chewing and suicidal ideation may partly reflect psychological distress and depressive symptoms associated with khat use, which may increase vulnerability to suicidal thoughts. Social and economic stressors associated with regular khat use may also contribute to this vulnerability (Damena et al., 2011; Olani et al., 2023).

The present study also showed that suicide attempts was significantly associated with the female sex. Females who use substances were 3 times more likely to attempt suicide as compared with male adolescents. This finding is supported by other studies done in the previous study in Ethiopia, Nigeria, Egypt, and Carolina (Adewuya & Oladipo, 2020; Bassiony et al., 2022; Garrison et al., 1993; Melkam et al., 2022b; Roy, 2003). Female adolescents may be more vulnerable to suicide attempts through interacting biological and psychosocial mechanisms, including pubertal changes, greater internalizing symptoms, and interpersonal stress. In substance-using high school students, psychological distress and impaired self-regulation may further increase vulnerability to suicide attempts (Miranda-Mendizabal et al., 2019b). However, contrary to this study, findings in South Africa show that suicide was more common in male than female adolescents (Khuzwayo et al., 2018).

Suicide attempts were also more common among substance users who have a comorbid chronic medical illness. The odds of having a suicide attempts were 6.4 times more common among students having known comorbid chronic medical illness as compared with their counterparts, which agrees with a study conducted among Nigerian adolescents (Adewuya & Oladipo, 2020). Substance users with comorbid medical illnesses may suffer from pain, disability, disfigurement, and an intrusive course of therapy, depending on their circumstances. These challenges may interfere with students' educational and leisure activities and may contribute to social isolation, which has been associated with suicidal behavior (Ferro et al., 2017).

The odds of suicide attempts were approximately three and a half times higher among substance-using high-school students who reported stress than among those who did not. Similar findings were reported in Belgium, Italy, and USA adolescents (Pompili et al., 2012; Portzky et al., 2005; Wilburn & Smith, 2005). Stressful life events, such as the death of a family member, academic difficulties, and substance-related stressors, may contribute to psychological distress and vulnerability to suicide attempts.

In the present study, substance-using students with a family history of suicide had two and a half times higher odds of reporting a suicide attempt than those without such a history. This finding is consistent with previous evidence showing that a family history of suicide is associated with an increased risk of suicidal behavior among adolescents and young people (Ferede et al., 2025; Tekeba et al., 2025). Suicide attempts is associated with an increased likelihood of suicide attempts among adolescents and young people. The elevated risk may reflect a combination of familial vulnerability, exposure to suicidal behavior within the family, shared adverse psychosocial environments, and behavioral modeling or imitation (Brent & Melhem, 2008). In substance-using high-school students these vulnerabilities may be further compounded by substance-related impaired judgment and reduced inhibition, potentially increasing susceptibility to suicidal behavior (Hua et al., 2023).

## 5 Limitations of the study

This study has several limitations. First, the cross-sectional design limits the ability to establish causal relationships between substance use, suicidal behavior, and associated factors. Second, the use of self-administered questionnaires may have introduced reporting and recall biases. Third, the small number of outcome events (18 suicide attempts and 30 suicidal ideation cases) limited the number of predictors that could be included in the multivariable models. Therefore, parsimonious models were used, with predictors selected based on clinical relevance and previously reported associations, and Firth penalized logistic regression was applied to reduce small-sample bias. Nevertheless, the limited number of events may have resulted in imprecise estimates and residual confounding. Fourth, potentially relevant variables, including perceived household income and bullying victimization, were not assessed and may have contributed to residual confounding. Finally, students were nested within seven schools, but the regression models were not adjusted for school-level clustering because of the small number of schools and outcome events. The findings should therefore be interpreted cautiously with respect to the precision and generalizability of the estimates. In addition, because the study included students aged 15–25 years enrolled in governmental high schools in three woredas, the findings may not be generalizable to private-school students, students in other areas, or adolescents and young people who are not enrolled in school.

## 6 Conclusion and recommendation

Overall, suicidal ideation and suicide attempts were reported by 7.4% and 4.4% of substance- using high-school students reporting lifetime use of alcohol, khat, or tobacco/cigarettes, respectively. Suicidal ideation was associated with depression symptoms, family history of suicide, and khat chewing, while suicide attempts were associated with female sex, family history of suicide, chronic medical illness, and stress. These findings highlight the importance of identifying depressive symptoms and stress and providing appropriate mental-health assessment, psychosocial support, and referral for students with identified needs. Attention should also be given to students with a family history of suicide, chronic medical illness, and khat use. Because of the cross-sectional design, these associations do not establish causality. Longitudinal and intervention studies are needed to determine whether addressing these factors can reduce suicidal behavior and substance-related harms among high-school students.

## CRediT authorship contribution statement

**Getasew Kibralew:** Writing – review & editing, Writing – original draft, Validation, Software, Methodology, Formal analysis, Data curation, Conceptualization. **Girum Nakie:** Writing – review & editing, Writing – original draft, Supervision, Investigation, Data curation. **Tesfaye Segon:** Supervision, Software, Methodology, Formal analysis.

## Consent for publication

Not applicable.

## Ethics approval and consent to participate

Ethical clearance was obtained from the Injibara University Ethical Review Committee on January 3, 2025 (Ref. No. IRERC/97/25). Permission was obtained from the respective Education Bureaus and participating schools. Written informed consent was obtained from participants aged ≥18 years, while parental/caregiver consent and student assent were obtained for participants aged <18 years. Participation was voluntary, and confidentiality and privacy were maintained throughout. Students reporting current suicidal risk were evaluated by the data collector and referred to the primary hospital in Yilmana Densa for further assessment and care. Relevant suicide-risk factors were communicated to the school, and the school psychologist was informed to facilitate follow-up support.

## Funding

This study received no specific funding from any public, commercial, or not-for-profit organization.

## Declaration of competing interest

The authors declare that they have no competing interests.

## Data Availability

The datasets generated and/or analyzed during the current study are available from the corresponding author upon reasonable request.
